# Monitoring targeted drug delivery: a key cornerstone of precision medicine

**DOI:** 10.1186/1479-5876-10-S2-A48

**Published:** 2012-10-17

**Authors:** György Marko-Varga, Thomas E Fehniger

**Affiliations:** 1 Div. Clinical Protein Science & Imaging, Biomedical Center, Dept. of Measurement Technology and Industrial Electrical Engineering, Lund University, BMC C13, SE-221 84 Lund, Sweden; 2 Dept. of Surgery, Tokyo Medical University, Tokyo, Japan

## 

The application of Personalized Medicine strategies of matching specific disease genotypes and phenotypes with specific pharmaceutical drug treatment has shown tremendous efficacy in several important diseases. The most successful example is the targeting mutations of the epithelial growth factor receptor by small molecule antagonists of the tyrosine kinase in non small cell lung cancer. The expansion of this treatment paradigm into other diseases has led to new thinking considering the diagnosis and treatment of disease, including “Precision Medicine”, which seeks to provide a molecular taxonomy of disease that can be used for guiding targeted therapy in individual patients. Today we have only a cursory understanding of the patterns of actual uptake of drugs or their active metabolites at such targeted sites in the body. In the past such studies required extrinsic labeling of the parent drug (ie radioactivity in PET) that potentially changed the chemical and biological properties of the drug. New technologies such as MALDI-MSI mass spectrometry have revolutionized the label-less tracking of drugs within targeted disease tissue sites [1]. A spectral image of drug distribution in biopsy tissue compartments is created by monitoring the signal intensity of signature ion mass fingerprints unique to each compound and its metabolites at contiguous sampling windows separated by < thirty microns. In a Proof of Principle study, we have applied MALDI-MSI to track the uptake and distribution of an inhaled muscarinic receptor antagonist [2], ipratropium in the bronchial airways of COPD patients shortly after administration. Direct measurement of the unlabeled drug in brochial biopsies showed that the ipratropium (parent drug ion mass, m/z 332.332, daughter ion masses m/z 166.2, and m/z 290.2) was transported and localized to areas of airway smooth muscle that expressed the targeted acetylcholine receptor M3, as shown by immunohistochemistry performed following mass spectrometry analysis. This result is the first reported co-localization of the unlabeled drug and its targeted receptor in man. In addition to the drug position and intensity signatures it is possible to simultaneously map the patterns of thousands of ion masses representing peptides, proteins, phospholipids, and metabolites that characterize healthy and diseased states. Further, the effect of drug on the relative abundances and histological positions of these ion signatures may also provide important indices of response to therapy. Together, these catalogs will likely become an important part of the molecular taxonomy of disease being developed with Precision Medicine frameworks using genomic, genetic and proteomic approaches defining the micro-environment of disease.
